# Experimental Dissection of Metalloproteinase Inhibition-Mediated and Toxic Effects of Phenanthroline on Zebrafish Development

**DOI:** 10.3390/ijms17091503

**Published:** 2016-09-08

**Authors:** Tonya R. Ellis, Bryan D. Crawford

**Affiliations:** Department of Biology, University of New Brunswick, Fredericton, NB E3B 5A3, Canada; tellis9@uwo.ca

**Keywords:** metalloproteinase, phenanthroline, metalloproteinase inhibitor, zebrafish, aryl hydrocarbon receptor, poly aromatic hydrocarbon toxicity, angiogenesis, neural crest, in vivo study

## Abstract

Metalloproteinases are zinc-dependent endopeptidases that function as primary effectors of tissue remodeling, cell-signaling, and many other roles. Their regulation is ferociously complex, and is exquisitely sensitive to their molecular milieu, making in vivo studies challenging. Phenanthroline (PhN) is an inexpensive, broad-spectrum inhibitor of metalloproteinases that functions by chelating the catalytic zinc ion, however its use in vivo has been limited due to suspected off-target effects. PhN is very similar in structure to phenanthrene (PhE), a well-studied poly aromatic hydrocarbon (PAH) known to cause toxicity in aquatic animals by activating the aryl hydrocarbon receptor (AhR). We show that zebrafish are more sensitive to PhN than PhE, and that PhN causes a superset of the effects caused by PhE. Morpholino knock-down of the AhR rescues the effects of PhN that are shared with PhE, suggesting these are due to PAH toxicity. The effects of PhN that are not shared with PhE (specifically disruption of neural crest development and angiogenesis) involve processes known to depend on metalloproteinase activity. Furthermore these PhN-specific effects are not rescued by AhR knock-down, suggesting that these are bona fide effects of metalloproteinase inhibition, and that PhN can be used as a broad spectrum metalloproteinase inhibitor for studies with zebrafish in vivo.

## 1. Introduction

Among metalloproteins, the metalloproteinases are often singled out as crucial mediators of extracellular matrix (ECM) remodeling in development and disease [1,2,3]. The matrix metalloproteinases (MMPs), a disintegrin and metalloproteinase domain containing proteins (ADAMs), and ADAMs with thrombospondin motifs (ADAMTSs) are evolutionarily related proteases containing a metzincin motif in their catalytic domain, so called because of the conserved methionine residue proximate to the zinc-binding catalytic site [3,4,5]. Best known for their roles in the hydrolysis of otherwise proteolytically resistant ECM proteins, especially during normal and pathological tissue remodeling, these proteases have more recently become recognized as essential components of signal transduction pathways—mediating the presentation of extracellular ligands, availability of cell surface receptors, etc.—and even intracellular processes [3,6,7,8,9,10].

Most of what we know about metalloproteinases is based on in vitro studies. The complexity of metalloproteinase regulation, and limitations of popular vertebrate model systems have made in vivo work especially difficult, and yet all the more important, as it is becoming increasingly clear that the tissue context in which these proteases function has enormous importance [11,12]. The zebrafish, long favored by developmental biologists and aquatic toxicologists, has emerged as a powerful vertebrate model in which to study the regulation of these proteases in vivo [13]. In addition to the availability of a wide variety of transgenic and mutant lines, the zebrafish is amenable to forward and reverse genetics, its genome is well characterized and can be manipulated using technologies such as CRISPR/Cas9, it develops rapidly and externally as a nearly transparent embryo allowing imaging of tissue remodeling and even protease activity in the living animal [14,15]. The zebrafish embryo has also recently become a favored model for the study of tumor cell behavior in xenografting experiments [16], increasing the importance of investigating the effects of compounds that may be relevant to the study of mammalian tumor cells in the context of zebrafish development.

**Figure 1:**
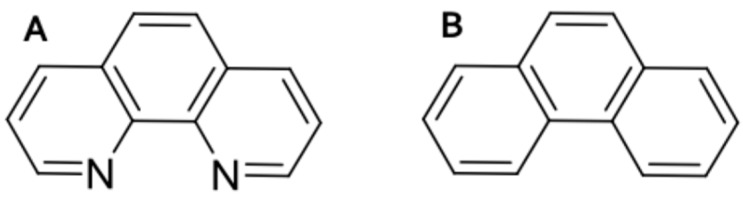
Phenanthroline (PhN) and phenanthrene (PhE) are structurally similar polyaromatic hydrocarbons (PAHs). Phenanthroline (**A**) is a tricyclic aromatic hydrocarbon with nitrogens at positions 1 and 10, which allow it to bind zinc, making it a potent inhibitor of metalloproteinases; Phenanthrene (**B**) is a classical PAH frequently used in the study of PAH-toxicity, but which has no known activity with respect to metalloproteinases.

Because of their central roles in tumor metastasis and other devastating pathologies, inhibitors of metalloproteinases have been the objects of intense investigation. In addition to natural inhibitors endogenous to tissues (e.g., tissue inhibitor of metalloprotenases (TIMPs), reversion-inducing-cysteine-rich protein with kazal motifs (RECK), α1 proteinase inhibitor, etc.), a wide variety of pharmacological inhibitors have been developed. These compounds fall largely into the categories of peptidomemetics, tetracycline derivatives, bisphosphonates, and non-specific zinc-chelators [17]. Among the latter, phenanthroline (PhN) is a tricyclic aromatic hydrocarbon with nitrogen atoms at positions 1 and 10, which allow it to bind zinc (Figure 1A). PhN is a broad spectrum metalloproteinase inhibitor with IC_50_ values in the µM range for a wide variety of these enzymes [18]. However, as a polyaromatic hydrocarbon (PAH) very chemically similar to phenanthrene (PhE) (Figure 1B), PhN is expected to have non-specific toxic effects, and is therefore rarely used in vivo.

PAH toxicity is mediated through the aryl hydrocarbon receptor (AhR). When AhR binds a PAH, it is targeted to the nucleus by dimerizing with the aryl hydrocarbon receptor nuclear translocator (ARNT) [19]. The activated AhR/ARNT dimer forms a transcriptional activator, triggering expression of cytochrome P450 family members. Thus, exposure to PAHs during early embryonic development results in high levels of cytochrome P450 expression [20]. In fish, this response is an adaptive mechanism for metabolizing the low concentrations of PAHs sometimes encountered in aquatic environments. However, the activity of cytochrome P450 results in the production of reactive oxygen species (ROS), so when PAHs are present at high concentrations the activity of this enzyme results in damage associated with high ROS levels [21].

Aquatic toxicologists have investigated the effects of PAHs on fish extensively [22,23,24]. PAH-toxicity often manifests as “blue-sac” disease, characterized by spinal curvature, pericardial edema, yolk-sac edema and reduced viteline circulation [25,26]. Morpholino knock down of AhR2 in zebrafish can reduce the induction of cytochrome P450 by PAHs, rescuing embryos from some aspects of PAH toxicity [27,28]. PhE binds the AhR, and is a well-characterized compound widely used in the study of PAH toxicity [29], and is also very chemically similar to PhN (Figure 1). We therefore reasoned that comparing the effects of PhE and PhN on the development of zebrafish, paying particular attention to processes thought to be dependent on metalloproteinase activity, combined with analyzing the protective effects (if any) of AhR2 knock-down, might allow us to dissect the metalloproteinase-inhibition-mediated and PAH-toxicity-mediated effects of PhN.

We show here that zebrafish embryos are notably more sensitive to PhN than PhE, and that PhN causes a superset of the effects of PhE. The effects common to PhE and PhN are characteristic of PAH toxicity and are at least partially rescued by a morpholino targeting AhR2. The effects of PhN not seen in embryos treated with PhE include disruptions of neural crest derived pigmentation and craniofacial development, and angiogenesis; processes thought to depend heavily on metalloproteinase activity. We therefore conclude that PhN has activities both as a metalloproteinase inhibitor and as a PAH, but that the latter can be largely avoided by using it at concentrations at or below 10 µM for in vivo experiments.

## 2. Results

### 2.1. Zebrafish Embryos Are More Sensitive to PhN than PhE

**Figure 2:**
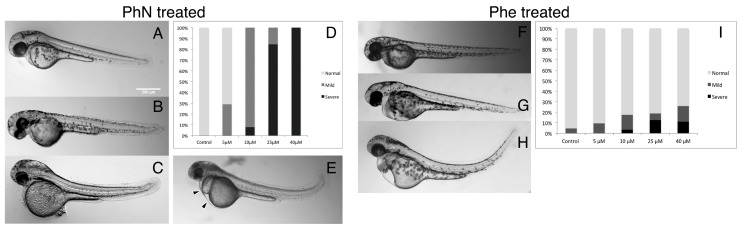
PhN and PhE both disrupt normal development, but embryos are more sensitive to PhN. Embryos were treated with either PhN or PhE at various concentrations from 24 to 48 hours post-fertilization (hpf), and assessed (blinded) for gross morphological defects visually. (**A**) A normal embryo from the vehicle control group. Embryos treated with PhN representing individuals scored as: (**B**) “Normal”; (**C**) “Mild”; and (**E**) “Severe” phenotypes (pericardial and yolk-sac edema indicated with arrowheads); (**D**) Summary of results from scoring 44 embryos exposed to vehicle only, 69 exposed to 5 µM PhN, 63 exposed to 10 µM PhN, 62 exposed to 25 µM PhN, and 88 exposed to 40 µM PhN. Embryos exposed to PhE exhibiting: (**F**) “Normal”; (**G**) “Mild”; and (**H**) “Severe” phenotypes; (**I**) Summary of results from scoring of 60 embryos exposed to vehicle only, 48 exposed to 5 µM PhE, 47 exposed to 10 µM PhE, 51 exposed to 25 µM PhE, and 40 exposed to 40 µM PhE. In addition to tail curvature, necrosis, pericardial and yolk-sac edema seen in embryos exposed to either PhE or PhN, embryos exposed to PhN exhibited loss of pigmentation, craniofacial abnormalities, and absence of intersomitic circulation. Scale bar = 200 µm.

We compared the effects of phenanthrene (PhE) and phenanthroline (PhN) on the development of zebrafish from 24 to 48 h post-fertilization (hpf) at various concentrations (Figure 2). As expected, both compounds perturb normal development, however PhN, which functions as a metalloprotinase inhibitor, is both quantitatively and qualitatively more potent than PhE; at 10 µM, the majority of embryos treated with PhE exhibited no obvious abnormalities, whereas all embryos treated with PhN at this concentration showed some effects. At 40 µM all PhN-treated embryos were severely affected, while most PhE-treated embryos still appeared normal. Furthermore, in addition to the effects characteristic of PAH toxicity exhibited by embryos treated with PhE (tail curvature, yolk-sac edema, and pericardial edema), embryos treated with PhN exhibited defects not seen in embryos treated with PhE at any concentration. The complete lack of pigmentation and craniofacial abnormalities are the most obvious of these, but other more subtle and potentially informative disruptions are discussed further below.

### 2.2. Loss of Pigmentation and Craniofacial Defects in PhN Treated Embryos Is due to Disruptions in Neural Crest Development

**Figure 3:**
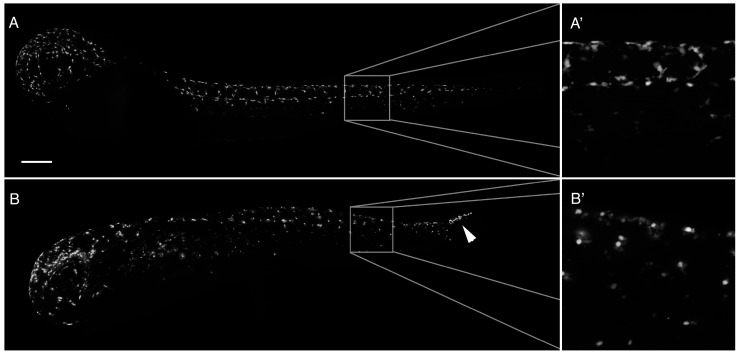
PhN perturbs development of neural crest-derived pigment cells. (**A**) A 48 hpf *Tg(mitfa:eGFP)* embryo exposed from 24 hpf to vehicle control, exhibiting normal distribution of GFP-expressing presumptive pigment cells. At high magnification (**A’**), the stellate morphology of these cells is apparent as they invade the overlying epidermis; (**B**) A 48 hpf *Tg(mitfa:eGFP)* embryo exposed from 24 hpf to 10 µM PhN, in which presumptive pigment cells in the head and anterior trunk have successfully emigrated from the neural tube and become distributed relatively normally, but in which presumptive pigment cells in the posterior tail have failed to emigrate from the neural tube (arrowhead). At high magnification (**B’**), these cells have a rounded amoeboid appearance. Boxes indicate regions magnified. Scale bar = 200 µm.

We speculated that the lack of pigmentation in PhN treated embryos might be due to disruption of neural crest-derived pigment cell emigration from the neural tube, their invasion of the overlying epidermis and/or their migration through the ECM; processes thought to depend on metalloproteinase activity [30,31,32,33,34]. To test this, we used *Tg(mitfa:eGFP)* embryos, in which the neural crest destined to produce pigment cells express green fluorescent protein [35]. When treated with 10 µM PhN the distribution of GFP-expressing presumptive pigment cells is significantly disrupted (Figure 3). When treated with 40 µM PhN this effect is even more profound (not shown). Interestingly, it appears those cells that had already emigrated from the neural tube before the introduction of PhN at 24 hpf are able to continue on their migration in the presence of the inhibitor, but cells at the posterior end of the neural tube that had not yet emigrated become trapped there when PhN is applied (Figure 3B, arrowhead). However, despite being able to migrate effectively, the presumptive pigment cells do not take on the characteristic stellate morphology of differentiated pigment cells in the presence of PhN (Figure 3, compare A’ to B’), and they appear to not invade the overlying epidermis.

**Figure 4:**
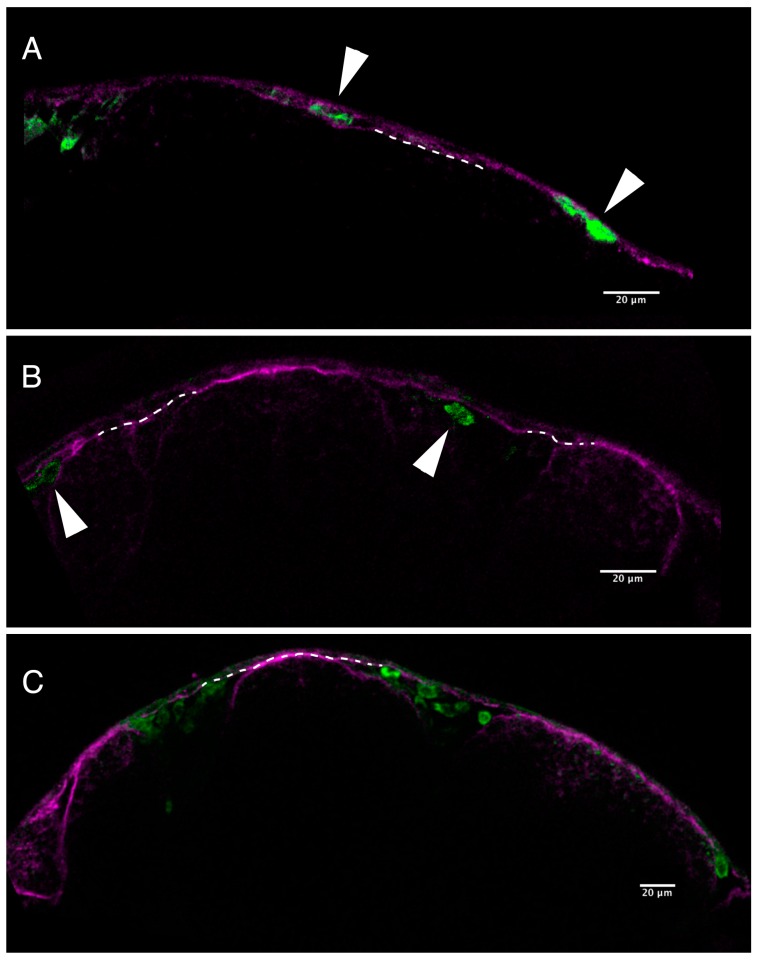
Pigment cells fail to invade epidermis in the presence of PhN. (**A**) A single focal plane through the head epidermis of a *Tg(mitfa:eGFP)* embryo exposed from 18 to 24 hpf to 10 µM PhE and stained with anti-laminin (magenta) and anti-GFP (green) showing presumptive pigment cells that have crossed the basal lamina and successfully invaded the epidermis (arrowheads); (**B**) A comparable single focal plane through the head epidermis of a *Tg(mitfa:eGFP)* embryo exposed from 18 to 24 hpf to 10 µM PhN, showing presumptive pigment cells (arrowheads) having failed to invade across the basal lamina of the epidermis; (**C**) A slightly lower magnification view of a *Tg(mitfa:eGFP)* embryo exposed from 18 to 24 hpf to 10 µM PhN, showing several presumptive pigment cells trapped below the epidermis. Dotted lines indicate the basal lamina. Scale bars are 20 µm.

To better visualize this failure of pigment cells to invade the overlying epidermis in the presence of PhN, we used an antibody against laminin to label the basal lamina underlying the epidermis (Figure 4). In embryos treated with 10 µM of the PAH PhE, presumptive pigment cells clearly invade the epidermis and are present between the basal lamina and the surface of the embryo (Figure 4A). However, in embryos treated with 10 µM PhN, this is not the case; GFP-expressing pigment cells do not cross the basal lamina (Figure 4B).

**Figure 5:**
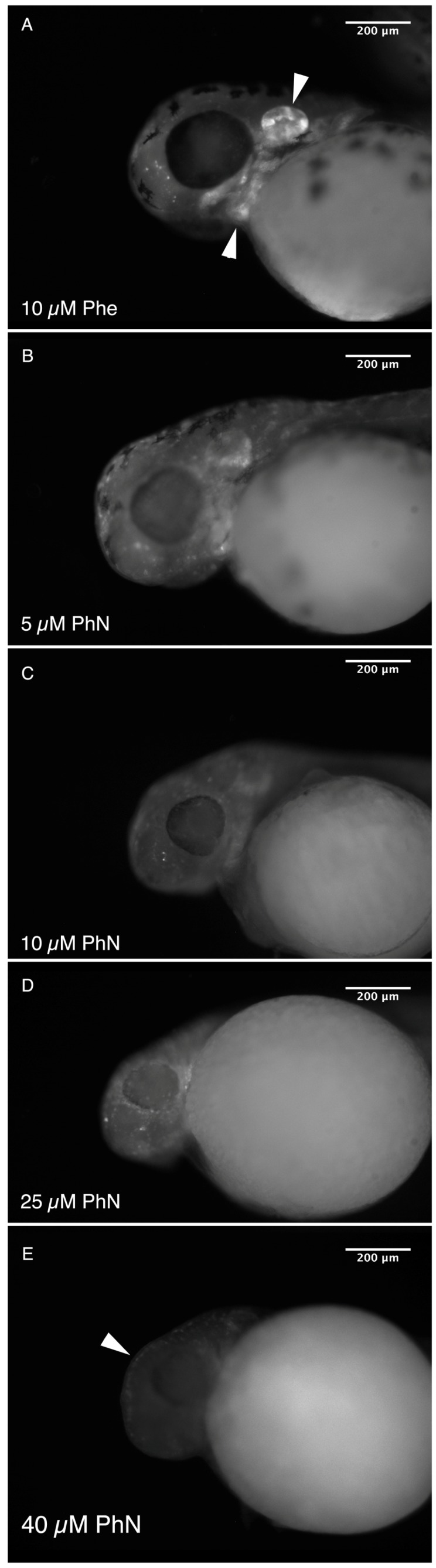
PhN disrupts craniofacial neural crest. (**A**) Epifluorescence micrograph of the head of a 48 hpf *Tg(sox10a:eGFP)* embryo treated from 24 hpf with 10 µM PhE showing normal development of the craniofacial neural crest (arrowheads indicate the otic vesicle and jaw cartilage). Embryos treated with: (**B**) 5; (**C**) 10; (**D**) 25; and (**E**) 40 µM PhN, exhibiting progressively more severe defects in these structures. Arrowhead in (**E**) indicates presumptive pigment cells trapped below the epidermis. Scale bars are 200 µm.

We note that PhN-treated (but not PhE-treated) embryos often exhibit abnormal crainofacial structure and/or disrupted otic vesicles. As these structures are also neural crest derived, we speculate that this effect of PhN may also be due to metalloproteinase-inhibition-dependent disruption of neural crest development. To investigate this further we treated *Tg(sox10a:eGFP)* embryos, which express GFP in all of their neural-crest derived cells [36], with various concentrations of PhN or PhE and examined the distribution of GFP-expressing neural crest cells in their heads (Figure 5). In *Tg(sox10a:eGFP)* embryos treated with 10 µM PhE, the distribution of GFP expressing cells is normal (Figure 5A), however in embryos treated with PhN, the GFP signal associated with the facial cartilages and otic vesicle is lost in a dose-dependent manner (Figure 5B–E). Consistent with our observations of *Tg(mitfa:eGFP)* cells failing to cross the basal lamina (Figure 4), presumptive pigment cells trapped beneath the ectoderm is also visible in *Tg(sox10a:eGFP)* embryos treated with PhN (Figure 5E, arrowhead).

### 2.3. Loss of Intersomitic Circulation in PhN Treated Embryos Is due to Disruptions in Angiogenesis

We noted the absence of visible intersomitic circulation in our 48 hpf PhN-treated embryos (not shown), and speculated that angiogenesis—a process known to be dependent on metalloproteinase activity [37,38]—might be disrupted in these embryos. We treated *Tg(fli1:eGFP)* embryos, which express GFP in their endothelial cells [39], with various concentrations of PhE and PhN between 24 and 48 hpf. For simplicity and consistency, we focused on the structure of the circulatory system of the trunk/tail when analyzing the effects of these experiments, as the development of the intersomitic vessels (ISVs) and dorsal longitudinal anastomosing vessel (DLAV) during this period of development has been very well-characterized [40].

**Figure 6:**
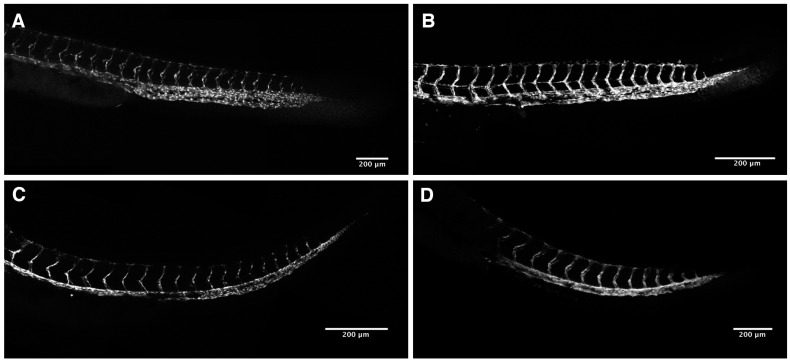
PhE does not disrupt angiogenesis. Forty-eight hpf *Tg(fli1:eGFP)* embryos treated from 24 hpf with: (**A**) vehicle alone; and (**B**) 10 µM; (**C**) 25 µM; or (**D**) 40 µM PhE exhibiting normal trunk vasculature.

**Figure 7:**
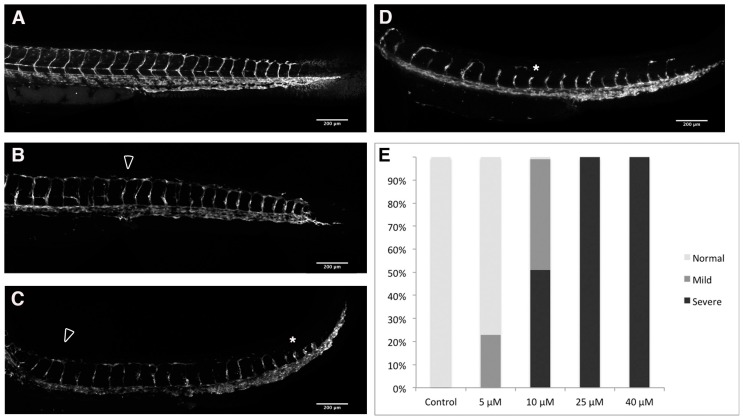
PhN disrupts angiogenesis. Forty-eight hpf *Tg(fli1:eGFP)* embryos treated from 24 to 48 hpf with either: (**A**) vehicle alone; or various concentrations of PhN (**B**–**D**), illustrating: (**B**) “Normal”; (**C**) “Mild”; and (**D**) “Severe” disruption of angiogenesis. The DLAV is indicated with an arrowhead in (**B**,**C**); and examples of disrupted angiogenesis are highlighted with asterisks; (**E**) Summary of blind scoring of 40 embryos exposed to vehicle alone, 96 embryos exposed to 5 µM PhN, 102 embryos exposed to 10 µM PhN, 91 embryos exposed to 25 µM PhN, and 77 embryos exposed to 40 µM PhN.

Embryos treated with the PhE, even at 40 µM, never exhibit disrupted angiogenesis in the trunk/tail (Figure 6), despite often exhibiting the characteristics of PAH toxicity discussed above (Figure 2). In contrast, even at very low concentrations, zinc-chelating PhN profoundly impairs the angiogenic sprouting of ISVs and their ability to form the DLAV (Figure 7). This effect shows a strong dose-responsiveness, with severe disruptions observable in all embryos treated with 25 µM PhN, and significant effects visible in nearly all embryos at less than half that concentration.

### 2.4. Effects Common to PhE and PhN Can Be Rescued by Reducing Expression of the Aryl Hydrocarbon Receptor

**Figure 8:**
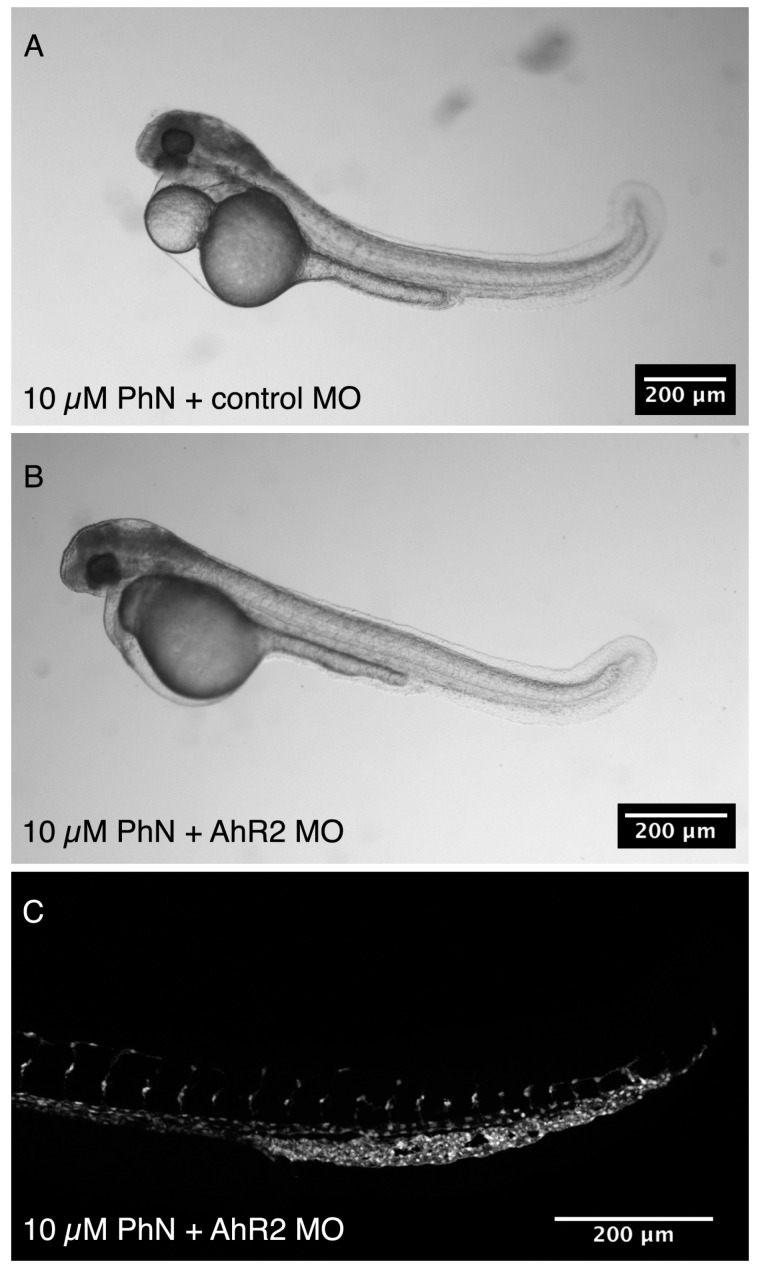
Morpholino knock-down of the aryl hydrocarbon receptor rescues some but not all effects of PhN treatment. *Tg(fli1:eGFP)* embryos were injected with morpholinos demonstrated to interfere with the expression of AhR2, thereby protecting them from PAH-toxicity, or control morpholinos, and were then exposed to 10 µM PhN from 24 to 48 hpf. (**A**) Control morphants exhibit typical PAH toxicity effects (tail curvature, necrosis, pericardial and yolk-sac edema), as well as disrupted angiogenesis, loss of pigmentation, and craniofacial defects; (**B**) Ahr2 morphants exhibit reduced pericardial and yolk-sac edema, but still exhibit loss of pigmentation, craniofacial defects; and (**C**) disrupted angiogenesis.

Thus, it appears that embryos treated with PhN exhibit effects of both metalloproteinase inhibition and PAH toxicity. To fully test this hypothesis, we used an antisense morpholino that has been shown to block expression of the aryl hydrocarbon receptor 2 (AhR2), and thereby protect embryos from PAH toxicity [27,28]. Effects of metalloproteinase inhibition should be unaffected by this manipulation, whereas effects of PAH-toxicity should be mitigated. *Tg(fli1:eGFP)* embryos were injected at the one-two cell stage with either the AhR2 morpholino (*n* = 62) or a control morpholino (*n* = 40), and allowed to develop to 24 hpf, at which point they were treated with 10 µM PhN until 48 hpf, at which time they were assessed (Figure 8). In all PhN-treated embryos pigmentation was completely abolished, and in all 50 individuals examined, significant disruptions in ISV development were apparent (Figure 8C). However, AhR2-morphants treated with PhN exhibited less pericardial and/or yolk-sac edema, and had less tail curvature than control-injected embryos (Figure 8B). Thus, the effects of PhN on neural-crest derived pigmentation and angiogenesis are not rescued by knock-down of AhR2, but the effects characteristic of PAH toxicity are.

## 3. Discussion

We examined the development of zebrafish embryos in the presence of a model poly aromatic hydrocarbon (PAH), phenanthrene (PhE), and the chemically-related zinc-chelating metalloproteinase inhibitor phenanthroline (PhN), focusing on differences in developmental processes thought to depend heavily on metalloproteinase activity in order to tease apart the PAH-toxicity-mediated and metalloproteinase-inhibition-mediated effects of PhN.

Most obviously, PhN was effective at much lower concentrations than PhE, and embryos exposed to PhN exhibit a superset of the effects we see in embryos exposed to PhE. As reported elsewhere, PhE treated embryos display defects characteristic of PAH-toxicity, often described as “blue-sac” disease, typified by upward tail curvature, yolk-sac and pericardial edema [25,26,41]. As a closely related chemical compound, it is not surprising that embryos exposed to PhN also exhibit these effects, and it is reasonable to suppose that this is occurring through the same mechanism. However, PhN-treated embryos also exhibit effects not observed in PhE-treated embryos, and we attribute these PhN-specific effects to its inhibition of metalloproteinase activity. Most compellingly, the effects of PhN we attribute to metalloproteinase inhibition are not rescued by morpholino knock-down of the aryl hydrocarbon receptor, whereas the effects of PhN we attribute to PAH toxicity are.

PhN appears to disrupt the development of neural crest-derived structures at two levels—the emigration of the crest cells from the neural tube (i.e., the epithelial-to-mesenchymal transition), and the invasion of crest cells into target tissues—resulting in embryos devoid of epidermal pigmentation (notably, retinal pigmentation is unaffected, demonstrating that melanin synthesis is unimpaired). We were unable to discern any effect on the rate or patterning of neural crest migration in time-lapse movies (data not shown), and presumptive pigment cells that had emigrated from the neural tube prior to the application of PhN appeared to be distributed normally (Figure 3), suggesting that metalloproteinase inhibition does not impair migration or pathfinding of the presumptive pigment cells. Pharmacological inhibition of MMP2 and 14 impaired melanophore migration in *Xenopus* [42], but broad spectrum metalloproteinase inhibitors did not affect neural crest migration in several other experiments [43]. Paradoxically, morpholino knockdown of Mmp17b alone significantly impairs neural crest migration in zebrafish [34] and knockdown of Mmp25a disrupts development of neural crest-derived neuromasts [44]. Furthermore, here we show that migration and/or survival of the neural-crest-derived cells of the craniofacial cartilages and otic vesicle is profoundly disrupted by PhN (Figure 4). These apparently contradictory results regarding the effect of broad-spectrum metalloproteinase inhibition on the migration of neural crest cells may be due to differences in the matrix the cells under investigation are migrating through; recent evidence suggest the porosity of the matrix may be an important trigger of protease expression by migrating cells [45], so migrating neural crest cells may need to employ these metalloproteinases only in specific contexts during their migration. Alternatively, it may be that these “broad spectrum” metalloproteinase inhibitors are more specific than generally believed; they may not effectively inhibit the specific metalloproteinases utilized by neural crest cells during their migrations in vivo.

PhN did clearly impact the ability of neural crest cells to emigrate from the neural tube, and invade into the epidermis, supporting the many previous reports of the importance of metalloproteinases in epithelial-to-mesenchymal transitions [30,32,46,47,48,49]. PhN also clearly prevented neural crest cells that had migrated from invading the overlying epidermis, further supporting the well-established role of metalloproteinases in invasiveness [50,51]. Interestingly, presumptive pigment cells that successfully migrated but were prevented from invading the epidermis in the presence of PhN did not take on their characteristic stellate morphology or produce melanin. This suggests that signals present in the epidermis, or some aspect of the process of invasion may act as a checkpoint in the differentiation of melanophores. The presence of melanizing signals in the epidermis of teleosts has been known for decades [52], but the underlying molecular mechanisms remain obscure. It seems plausible that invasion into the epidermis alters the availability of signals, possibly consisting of Wnt5a [53], and/or signals derived from intact or degraded ECM molecules such as laminin [54] or elastin [55], which in turn impinge on the regulation of Sox5 [56] and/or other regulators of melanophore differentiation.

The importance of metalloproteinases in the development of the neural-crest-derived craniofacial structures has also been well established [43,57], supporting our attribution of the effect of PhN on craniofacial development to metalloproteinase inhibition. The effects of PhN on angiogenesis are also most parsimoniously explained as the result of metalloproteinase inhibition, as the sprouting, invasive protrusion and pathfinding activities of angioblasts all depend on metalloproteinases [37,58,59,60]. Thus, we conclude that the effects of PhN on angiogenesis, pigmentation and craniofacial development are most likely due to its inhibition of metalloproteinases, whereas tail curvature, pericardial and yolk-sac edema are likely manifestations of PAH-toxicity.

The effects of PhN mediated by metalloproteinase inhibition become detectable at concentrations as low as 5 µM, and by 10 µM nearly 100% of embryos exhibit defects in angiogenesis and neural crest development. At these concentrations, PhE has very little effect, and the PAH-toxicity effects of PhN are not severe. PhN is rarely used as a metalloproteinase inhibitor in vivo because of its presumed off-target toxic effects, but we show here that at concentrations below 10 µM, these off-target effects are minimal, whereas impacts on metalloproteinase-mediated processes are significant. Furthermore, the PAH toxicity effects of PhN appear limited to causing yolk-sac and/or pericardial edema and tail curvature. If researchers using zebrafish embryos are aware of this, we argue that PhN can be used in vivo with caution.

## 4. Materials and Methods

### 4.1. Zebrafish Husbandry

Zebrafish (*Danio rerio*) embryos were obtained by natural spawning over marbles from Tübingen (wild-type), *Tg(mitfa:eGFP)* (expressing GFP in presumptive pigment cells), *Tg(sox10:eGFP)* (expressing GFP in all neural crest derived cells), or *Tg(fli1:eGFP)* (expressing GFP in endothelial cells) fish (originally obtained from the Zebrafish International Resource Center (ZIRC) at the University of Oregon) maintained on a 14 h light, 10 h dark cycle (as described by [61]) in the University of New Brunswick Zebrafish Facility. Embryos were reared at 28 °C and staged according to [62]. All procedures involving animals were approved and monitored by the UNB Animal Care Committee, under Animal Use Protocols 14014 (23 April 2014), 15016 (7 May 2015), and 16018 (28 April 2016), according to the standards of the Canadian Council on Animal Care.

### 4.2. Phenanthrene (PhE) and Phenanthroline (PhN) Treatments

100 mM stock solutions of PhE and PhN in DMSO (Sigma, Oakville, ON, Canada) were diluted to 5, 10, 25, or 40 µM (the highest concentration at which PhE could be dissolved) in embryo rearing medium (ERM) (130 mM NaCl, 0.5 mM KCl, 0.02 mM Na_2_HPO_4_, 0.04 mM KH_2_PO_4_, 1.3 mM CaCl_2_, 1.0 mM MgSO_4_, 0.4 mM NaH_2_CO_3_, pH 7.4) at 28 °C and vortexed extensively. Embryos were incubated in clean ERM until the specified stage (usually 24 hpf), then manually dechorionated using fine forceps and transferred into dishes with 50 mL of either the specified concentration of PhE or PhN, or vehicle control (20 µL DMSO in 50 mL ERM, making the [DMSO] comparable 40 µM treatments) and incubated for the specified time at 28 °C in a darkened humidified incubator.

### 4.3. Scoring of Embryonic Defects

Before scoring, dishes containing treated embryos were randomized and assigned an arbitrary letter by a colleague not involved with the experiments; all scoring was done “blind” (i.e., without knowledge of the treatment the embryos had been exposed to), and results of scoring assigned to a treatment group *ex post facto*.

Morphological defects associated with “blue-sac disease” (tail curvature, pericardial, yolk-sac edema, and necrosis) were scored using a Leica M205FA stereo microscope (Leica-microsystems, Concord, ON, Canada). Embryos were assigned a grade of “Normal” (exhibiting none of these effects), “Mild” (exhibiting only one or two of these effects), or “Severe” (exhibiting all of these effects). Only living embryos (i.e., with a visible heart beat) were scored.

Defects in angiogenesis were scored on embryos that were fixed in 4% formaldehyde (diluted in ERM) for 2 h at room temperature at the end of the treatment time (in order to eliminate variation in the timing of scoring between embryos) and then imaged using a Leica M205FA stereo epifluorescent microscope equipped with excitation/emission filters optimized for GFP. Embryos were scored as “Normal” if there were three or fewer incomplete ISVs (i.e., ISVs that had failed to extend to the dorsal limit of the somite, where they form a “T” junction with the DLAV), “Mild” if there were between 3 and 10 incomplete ISVs, and “Severe” if there were over 10 incomplete ISVs.

### 4.4. Immunofluorescence

Embryos were fixed in 4% formaldehyde diluted in ERM overnight at 4 °C, washed 3 × 15 min in phosphate buffered saline with 0.1% Triton X-100 (Sigma) (PBSTx), then blocked overnight at 4 °C in PBSTx + 5% bovine serum albumin (Thermo, Ottawa, ON, Canada). Blocked embryos were incubated in primary antibodies (rat-anti-GFP and rabbit-anti-laminin) diluted 1:500 in blocking buffer overnight at 4 °C. Unbound primary antibodies were removed by washing 3 × 15 min in PBSTx, followed by incubation in goat-anti-rat-Alexa594 and goat-anti-rabbit-Alexa488 secondary antibodies diluted 1:1000 in blocking buffer for 2 h at RT. Unbound secondaries were removed by washing 3 × 15 min in PBSTx at 37 °C, before mounting and imaging as described below.

### 4.5. Morpholino Injections

*Tg(fli1:eGFP)* embryos were collected immediately after spawning and injected at the 1–2 cell stage with 3–5 nL of 100 µM AhR2 or Control morpholino, diluted in Danieau buffer (58 mM NaCl, 0.7 mM KCl, 0.4 mM MgSO_4_, 0.6 mM Ca(NO_3_)_2_, 5 mM HEPES, pH 7.6). Control morpholino with the sequence 5′-GACGTTGTCATTTATTTGATTTTCG-3′ was purchased from GeneTools, LLC., Philomath, OR, USA, and is reported to have no detectable effects on zebrafish development (GeneTools). AhR2 morpholino with the sequence 5′-TGTACCGATACCCGCCGACATGGTT-3′ was a generous gift of Dr. Teraoka, and has been demonstrated to effectively block expression of the aryl hydrocarbon receptor in zebrafish [27,28]. Morpholino injected embryos were allowed to develop for 24 h at 28 °C in ERM, before being manually dechorionated and transferred to dishes with 10 µM PhN or vehicle controls. Morphant embryos were exposed to these treatments for 24 h before being examined for morphological defects and fixed in 4% formaldehyde and imaged by confocal microscopy.

### 4.6. Imaging and Image Processing

Morphological characterization and epifluorescent imaging of defects in angiogenesis (in *Tg(fli1:eGFP)* embryos) or craniofacial crest development (in *Tg(sox10:eGFP)* embryos) was done using a Leica M205FA stereo microscope with a DFC360 FX camera and GFP excitation/emission filters. Immunofluorescence assays and high resolution images of pigment cells in *Tg(mitfa:eGFP)* embryos and the tail vasculature in *Tg(fli1:eGFP)* embryos were imaged using a Leica SP2 confocal microscope (Leica-microsystems, Concord, ON, Canada) fitted with 20 × 0.7 NA and 63 × 1.4 NA water immersion lenses. Addition of scale bars, and minor adjustments to brightness/contrast were done using in Fiji [63] and high-resolution composite images assembled using the pair-wise stitching plug-in. Figures were assembled and annotated using Photoshop CS 6 (Adobe, San Jose, CA, USA).
